# Dendritic cell-specific transmembrane protein is required for synovitis and bone resorption in inflammatory arthritis

**DOI:** 10.3389/fimmu.2022.1026574

**Published:** 2022-11-07

**Authors:** Maria de la Luz Garcia-Hernandez, Javier Rangel-Moreno, Maricela Garcia-Castaneda, H. Mark Kenney, Ananta Paine, Michael Thullen, Allen P. Anandarajah, Edward M. Schwarz, Robert T. Dirksen, Christopher T. Ritchlin

**Affiliations:** ^1^ Division of Allergy/Immunology and Rheumatology, University of Rochester, Rochester, NY, United States; ^2^ Department of Pharmacology and Physiology (SMD), University of Rochester Medical Center, Rochester, NY, United States; ^3^ Center for Musculoskeletal Research. University of Rochester, Rochester, NY, United States

**Keywords:** DC-STAMP, arthritis mouse model, type 2 cytokines, TNF, CCL2

## Abstract

**Objective:**

Dendritic Cell-Specific Transmembrane Protein (DC-STAMP) is essential for the formation of fully functional multinucleated osteoclasts. DC-STAMP deficient mice, under physiological conditions, exhibit osteopetrosis and develop systemic autoimmunity with age. However, the function of DC-STAMP in inflammation is currently unknown. We examined whether genetic ablation of DC-STAMP attenuates synovitis and bone erosion in TNF transgenic (Tg) and K/BxN serum-induced murine rheumatoid arthritis.

**Methods:**

We evaluated arthritis onset in Tg(hTNF) mice lacking DC-STAMP and 50:50 chimeric mice by visual examination, measurement of ankle width, micro-CT-scan analysis and quantitation of the area occupied by osteoclasts in bone sections. To further investigate the cellular and molecular events modulated by DC-STAMP, we measured serum cytokines, determined changes in cytokine mRNA expression by monocytes activated with IL4 or LPS/IFNγ and enumerated immune cells in inflamed mouse joints.

**Results:**

Synovitis, bone loss and matrix destruction are markedly reduced in Dcstamp^-/-^;Tg(hTNF) mice. These mice had significantly lower CCL2 and murine TNF serum levels and exhibited impaired monocyte joint migration compared to Tg(hTNF) mice. The reduced arthritic severity in Dcstamp deficient mice was associated with compromised monocyte chemotaxis, cytokine production, and M2 polarization.

**Conclusion:**

These results reveal that DC-STAMP modulates both bone resorption and inflammation and may serve as an activity biomarker and therapeutic target in inflammatory arthritis and metabolic bone disease.

## Introduction

Multinucleated osteoclasts orchestrate pathologic bone loss, a dynamic process operative in inflammatory arthritis, osteoporosis, and metastatic disease (1). The differentiation of monocyte progenitors into osteoclasts initially relies on the Receptor of NFkB (RANK) and RANK ligand (RANKL) interaction, resulting in enhanced NFATc1-dependent activation of TRAF6 and c-Fos pathways (2–4). Translocation of NFATc1 from the cytoplasm to the nucleus, triggered by de-phosphorylation *via* the Ca^+2^/calmodulin-dependent phosphatase calcineurin, initiates the transcription of genes (i.e., *Atp6vd2* and *Dcstamp*) that result in the programmed differentiation of mononuclear monocytes into a polykaryon that degrades bone matrix (5–8). Sustained activation of NFATc1 is mediated by low-level calcium signaling required during the entire course of osteoclastogenesis (9). A key event in this process is cell-cell fusion directed by the master regulator Dendritic-Cell Specific Transmembrane Protein (DC-STAMP), present on the surface of osteoclast precursors (6).

DC-STAMP, initially discovered in the endoplasmic reticulum of dendritic cells, is found on the cell membrane of monocytes (10, 11). While NFATc1 induces DC-STAMP expression soon after RANKL/RANK engagement, we demonstrated that DC-STAMP regulates NFATc1 degradation and nuclear translocation from the cytoplasm to the nucleus by modulating low-level Ca^+2^ signaling (12), essential for multinucleation, differentiation, and osteoclast (OC) function. Interestingly, myeloid progenitor cells isolated from Dcstamp^-/-^ mice and activated *in vitro* with M-CSF and RANKL only formed mononuclear OCs with limited capacity to resorb bone pits. Consistent with the compromised ability of DC-STAMP deficient OCs to resorb bone, mild osteopetrosis manifests in Dcstamp deficient mice, while DC-STAMP over-expressing transgenic mice exhibit severe osteoporosis (11, 13–15).

The function of DC-STAMP is not limited to bone homeostasis. For example, aged DC-STAMP knockout mice exhibit autoimmune features presenting as spontaneous lymphoproliferation and increased production of autoantibodies specific for double stranded DNA (16). Dendritic cells from these mice showed enhanced phagocytic activity and T cell priming supporting the view that DC-STAMP regulates dendritic cell phagocytosis and self-tolerance. In contrast, knock down of DCSTAMP in bone marrow dendritic cells (BMDCs) reduces secretion of cytokines (IL-6, TNF, IL-10, and IL-12), increases IL-1 levels, and impairs T cell activation (17). Direct inhibition of DC-STAMP with the MiR-7b microRNA disrupts NFATc1-Ca^2+^ signaling in osteoclasts and blocks the synthesis of key osteoclastogenic factors (NFATc1, osteoclast-associated receptor; OSCAR) and a number of molecules involved in cell-cell fusion, including CD47, Atp6vd2, and the Osteoclast-Specific Transmembrane Activating Protein (OC-STAMP) (18).

Given that NFATc1 serves as a master regulator in OC formation, inflammation, angiogenesis, cartilage catabolism, and pain, we hypothesize that deletion of DC-STAMP would greatly reduce inflammation. Herein, we examined the role of DC-STAMP deficiency on joint inflammation and bone damage in the tumor necrosis factor transgenic (Tg(hTNF)) mouse model of chronic inflammatory-erosive arthritis and the K/BxN serum transfer model of acute joint inflammation. In both chronic and acute inflammatory arthritis, the significant reduction in synovitis and joint damage in Dcstamp^-/-^ mice underscores the importance of this molecule in regulating inflammation and pathologic bone damage during inflammatory arthritis.

## Materials and methods

### Mice

We obtained C57BL/6, B6.CD45.1 and B6.H2^g7^ mice from Jackson laboratory and Toshio Suda (Keio University) kindly provided DCSTAMP*^-/-^* mice. The 3647-tumor necrosis factor transgenic (Tg3647) mouse line was originally obtained from Dr. George Kollias (19, 20), and has been maintained across multiple generations in a C57B/6 background. This line harbors a modified human TNF transgene in which the 3’-region was replaced with a human β-globin gene. All studies were performed with littermate wild-type controls (WT). We obtained B6.KRN mice from Dr. Diane Mathis (Harvard University). We generated Dcstamp^-/-^; Tg(hTNF) mice and Dcstamp^-/-^ CD45.1 in our laboratory. Mice were bred and maintained in the animal facility at the University of Rochester, and the University of Rochester Committee on Animal Resources approved all experimental protocols (102265). We analyzed bone resorption and/or immune response in 5-month-old female Tg(hTNF), Dcstamp^-/-^; Tg(hTNF), Dstamp^-/-^ or C57BL6 mice by histology, ELISA, LEGENDPlex and quantitative PCR.

### Bone marrow chimeras

We irradiated 7-8-week-old C57BL/6 mice with divided two doses of 950 Rads (475 Rad each), using an irradiator with a 137Cs source and infused with a mixture of BM to generate 50:50 chimeras. Irradiated recipient mice were reconstituted, *via* retro-orbital injection, with 1 x 10^7^ bone marrow cells collected from CD45.1 Dcstamp^-/-^ and CD45.2 Tg(hTNF) mice. We isolated blood leukocytes by gradient density using Lympholite M (Cedarlane, Burlington, CA) and stained them with specific antibodies against CD45.2 and CD45.1 (Biolegend, San Diego, CA.) to verify 50:50 bone marrow cells reconstitution. 6-weeks after bone marrow reconstitution, we injected the mice at days 0 and 3 with 150 μl of serum collected from 8-week-old K/BxN mice. Twelve days after serum transfer, we euthanized the animals, and their hind paws were harvested for histology analysis or injected with an additional 150 µl of K/BxN serum on day 26 and sacrificed on day 30 for μCT analysis.

### OCP differentiation

We cultured 1 x 10^5^ bone marrow cells, isolated from 7–8-week-old WT, Dcstamp^-/-^, Tg(hTNF) and Dcstamp^-/-^;Tg(hTNF) mice, in a 96-well plates in alpha - DMEM media supplemented with 10 μg/mL of M-CSF (415-ML, R&D system, Minneapolis., MN) 2 mM L-glutamine, 100 μg/mL streptomycin, 1 mM of non-essential amino acids, 1 mM sodium pyruvate, and 0.5 μM of amphotericin B. On day three, fresh media containing 10 μg/mL of M-CSF and 10 μg/mL of RANKL was added (462- TEC, R&D system, Minneapolis., MN) and cell cultures were maintained for additional four days. Cells were then fixed with citrate/37% of formaldehyde solution. Osteoclast differentiation was evaluated with a TRAP activity kit according to the manufacturer’s instructions (387A, Sigma-Aldrich Co LLC. St Louis., MO).

### Isolation of splenic macrophages

8-week-old WT, Dcstamp^-/-^, Tg(hTNF), and Dcstamp^-/-^; Tg(hTNF) female mice were injected intraperitoneally with 10 μg of LPS (055: B5, Sigma-Aldrich Co LLC, St Louis, MO) to activate immune cells *in vivo*. 24-hours after LPS injection, spleens were harvested, and cell suspensions were incubated with 2 μg/ml 2.4G2 for 10-minutes at 4oC to block Fc receptor binding. Cells were washed once and then incubated with biotin-conjugated CD11b antibodies (101203, clone M1/70, Biolegend, San Diego, CA.) for 15-minutes at 4°C, followed by incubation with streptavidin coupled to magnetic beads and passed through an LS+ column (130- 042-041, Miltenyi Biotech Auburn, CA.). We incubated CD11b positive cells at 37°C for 30 min to remove non-adherent cells. Adherent cells were cultured in complete RPMI1640 media containing 20 ng/ml of IL-4 and 30 ng/ml of M-CSF (214-14, 315-02 Peprotech, Rocky Hill., NJ) to generate M2 macrophages. We incubated adherent cells for 6-hours with complete RPMI1640 media containing 25 ng/ml of murine IFNγ (315-05, Peprotech, Rocky Hill, NJ) followed by the addition of 100 ng/ml of LPS (055: B5, Sigma-Aldrich Co LLC, St Louis., MO.) to generate M1 macrophages. We harvested macrophages after 16-hours of incubation with IL-4/M-CSF (M2) or IFNγ/LPS (M1), and RNA was extracted to assess gene expression. Cytokine and chemokine secretion; 2 x10^6^ cells were cultured for 72 hr at 37°C 5% of CO_2_ in a 6-well plate with 3 ml of complete RPMI media. We measured cytokine and CCL2 release with LEGENDplex™ inflammation panel assay, according to the manufacturer’s instructions (Biolegend, San Diego, CA.).

### Micro-CT analysis

Micro-CT scans (Scanco Medical, Viva CT 40 cone-beam CT, Switzerland) were performed on femurs and tibias collected from each experimental group of mice. Bones were fixed for 48-hours with 10% formalin, washed twice with PBS and placed into the imaging tube of a VivaCT 40. Segments were scanned with 55 kVp intensity, a current of 145 micro-amps and 1000 projections over 1800 during 300-ms integration time, producing a resolution of 10.5-micron isotropic (cubical) voxel size. Femur trabecular bone analysis was initiated at the proximal end of the growth plate and proceeded proximally another 100 slices (10.5-micron slices). Tibia trabecular analysis started at the distal end of the growth plate and proceeded 100 slices distally. Analysis of 3-dimensional images obtained using a Gaussian filter (s = 0.8, Gauss support = 1.0) with simple segmentation at Scanco threshold of 310 (2.480cm-1) were performed using a Scanco evaluation software V6.5.

### Histology and immunofluorescence

We fixed knee and ankle joints in 10% formalin for 48-hours and then decalcified them in 14% of EDTA for 2-weeks before embedding them in paraffin. Then, 4 μm sections were stained with either: 1) Alcian Blue Hematoxylin/Orange G or 2) TRAP to detect OC activity. Pannus area and the percentage of bone area occupied by OCs in femurs and tibias was measured using the outline tools in a Zeiss AxioCam digital camera (Carl Zeiss, San Diego, CA) and ImageJ software (NIH). For immunofluorescence, we blocked sections with 10 μg/ml 2.4G2 and 5% donkey serum in PBS for 30-minutes at room temperature. We blocked endogenous biotin activity with a commercial biotin-avidin kit (SP-2001, Vector Laboratories, Burlingame, CA) followed by incubation with biotin-conjugated CD11b/c (NB110-40766, Novus biological, CO), CD3e (clone M-20; Santa Cruz Biotechnology, Dallas, Tx), biotin-conjugated TNF (NBP1-19532, Novus Biologicals, CO) antibodies overnight at room temperature. We detected primary antibodies with DyLight 649-conjugated streptavidin (SA-5649-1, Vector laboratories, Burlingame., CA), and Cy3-conjugated donkey anti-goat IgG (705-166-147, Jackson ImmunoResearch Laboratories, West Grove, PA) or AF647 donkey anti-rabbit antibodies (111-606-152, Jackson ImmunoResearch Laboratories, West Grove, PA) for 1-hour at room temperature. Slides were washed and mounted with Vectashield mounting medium (H-1500, Vector laboratories, Burlingame., CA). Representative images were taken with a Zeiss Axioplan Microscope and recorded with a Hamamatsu Camera.

### Measurement of serum cytokines and quantitative PCR

Following sacrifice, we collected blood from the renal vein of C57BL/6, Dcstamp^-/-^, Tg(hTNF) and Dcstamp^-/-^;Tg(hTNF) mice and centrifuged the blood at 1500 r.p.m for 10 minutes. We measured murine TNF and CCL2 using LEGENDPlex bead-based immunoassay according to the manufacturer’s instructions (740446, Biolegend, San Diego., CA). We calculated cytokine concentration using the LEGENDPlex data analysis software. Human TNF was measured by an ELISA kit purchased from Invitrogen, and the assay was performed according to the manufacturer’s instructions (88-7346-22, Thermo Fisher Scientific, Grand Road., NY). We isolated RNA from macrophages using an RNAeasy plus mini kit (Cat # 74034, Qiagen, Germantown., MD). RNA was reverse transcribed using a high-capacity cDNA reverse transcription kit (4366814, Thermo Fisher Scientific, Grand Road., NY), and quantitative PCR was performed on a Quantstudio 3 real-time PCR system (Thermo Fisher Scientific, Grand Road., NY) using specific probes and TaqMan Universal Master Mix (440038, Thermo Fisher Scientific, Grand Road., NY) according to the manufacturer’s instructions. We normalized the relative mRNA expression to GAPDH and then normalized to the expression of WT M0 macrophages.

### Flow cytometry

Spleen, popliteal lymphoid nodes and synovial cell suspensions from C57BL/6, Dcstamp^-/-^, Tg(hTNF) and Dcstamp^-/-^;Tg(hTNF) mice were incubated in PBS containing 1% of heat-inactivated FBS (FACS media) (12550H, Thermo Fisher Scientific, Grand Road NY), 2 mM of EDTA and 10 µg/ml of Fcblock (clone 2.4G2, BioXcell, Lebanon, NH) for 10-minutes on ice and stained with CD11b-APC/Cy7 (clone M1/70), CD64-APC (clone X54-5/7.1), CD45-PEcy7 (clone 30-F11) or CD3 APC/Cy7(clone 500A2), Cells were fixed in 4% paraformaldehyde, permeabilized and finally incubated with CCL2-PE (clone 2H5) and TNF-FITC (clone MP6-XT22), in permeabilization buffer for 30-minutes on ice. Cells were washed twice and resuspended in FACS media. For bone marrow chimeras, peripheral blood leukocytes were stained with anti CD45.2-percpCy5.5 (clone A20) CD45.1-AF700 (clone 104), CD64 APC (clone X54-5/7.1), CD11b-APC Cy7 (clone clone M1/70), CCR1 FITC (clone S15040E), CCR2-PE (clone SA203G11), CX3CR1-PEcy7 (clone SA011F11). Cell acquisition was performed on an LSRII cytometer (BD Biosciences, San Jose. CA) and data were analyzed with FlowJo software (Treestar, Ashland, OR). All the flow cytometry antibodies were purchased from Biolegend, San Diego, CA.

### Statistical analysis

We used GraphPad Prism software (version 9.0) for all statistical analyses. Shapiro-Wilk normality *test* and Two-way ANOVA was used to compare among groups. Tukey’s multiple comparisons, Student’s t-test or Mann-Whitney test for two group comparison. Results are expressed as mean ± SD and p ≤ 0.05 was considered significant.

## Results

### Synovitis and matrix destruction are markedly reduced in Dcstamp^-/-^;Tg(hTNF) mice

**Figure 1:**
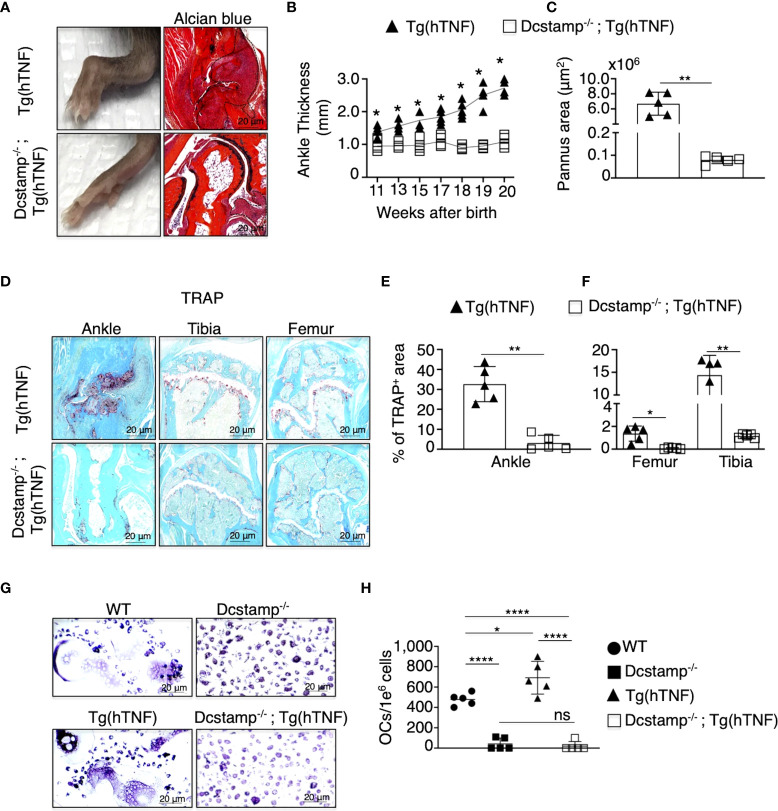
Inflammatory arthritis and pathologic bone resorption are markedly decreased in Tg(hTNF) mice with genetic deletion of Dcstamp. **(A)** Representative photographs of the hind paw and ankle joint from 5-month-old Dcstamp^-/-^; Tg(hTNF) and Tg(hTNF) female mice stained with Alcian Blue. Tg(hTNF) mice have visibly swollen ankles and marked synovial inflammation; findings not observed in Dcstamp^-/-^; Tg(hTNF) mice. **(B, C)** show significant differences in ankle thickness and pannus area in the Tg(hTNF) vs. Dcstamp^-/-^; Tg(hTNF) mice. n = 5 (one back paw/mouse), mean ± SD. p=0.05 (two-way ANOVA and Tukey's multiple comparison test.) **(D–F)**. Morphometric analysis shows significant reduction in the TRAP+ area of the ankle, tibia and femur of Dcstamp^-/-^; Tg(hTNF) vs. Tg(hTNF) mice. 200x magnification pictures captured with a Zeiss Axioplan microscope and recorded with a Hamamatsu camera **(D**, **G)**. Scale bars represent 20 um. The area covered by TRAP+ osteoclasts in mouse ankle, tibia and femur was analyzed with NIH Image J software. Graphs represent the mean ± SD (n = 5 mice). ns, not statistically significant, *p < 0.05, **p < 0.005 (Student's t-test). **(G)** Bone marrow cultures from WT and Tg(hTNF) mice incubated under osteoclastogenic conditions, showed differentiation of large and multinucleated osteoclasts than DC-STAMP deficient mice. **(H)** The number of *in vitro* differentiate multinucleated OCs was compared in Dcstamp^-/-^ and Dcstamp^-/-^;Tg(hTNF) Bone Marrow Cells (BMC). Representative pictures of the number of osteoclasts differentiated in vitro are shown, n=5, mean ± SD. *p ≤ 0.05, ****p ≤ 0.00005. (two-way-ANOVA and Tukey's multiple comparison test).

In previous studies performed under physiological conditions, DC-STAMP was critical for the generation of multinucleated OCs (13). However, the overall function of DC-STAMP in the context of an inflammatory arthritis model is unknown. To examine the impact of DC-STAMP deficiency in arthritis, we crossed Dcstamp^-/-^ mice to Tg(hTNF) mice, a model characterized by the development of chronic inflammatory-erosive arthritis (21). We assessed the progression of joint disease in Dcstamp^-/-^;Tg(hTNF) and Tg(hTNF) mice by evaluating joint swelling along with histopathological and imaging findings in the ankles, tibias, and femurs. The Dcstamp^-/-^;Tg(hTNF) compared to the Tg(hTNF) mice, demonstrated significantly less joint swelling and cartilage loss, and the inflammatory cell infiltration and pannus formation were markedly reduced in the absence of DC-STAMP (**Figures 1A–C**). The percentage of the TRAP^+^ area in the ankles of Dcstamp^-/-^;Tg(hTNF) compared to the Tg(hTNF) mice was (4.9% vs 36%) in the tibia (1.05% vs 17.3%) in the femur (0.1% vs 0.4%) (**Figures 1D–F**). We then examined the ability of monocytes isolated from the Dcstamp^-/-^;Tg(hTNF) mice to form multinucleated OCs in the setting of TNF-driven inflammatory arthritis. OC progenitors from the bone marrow of Dcstamp^-/-^;Tg(hTNF) mice were cultured *in vitro* to induce OC differentiation. We found a significant reduction in the number of multinucleated OCs in cultures of bone marrow cells isolated from both Dcstamp^-/-^ and Dcstamp^-/-^;Tg(hTNF) mice compared to similarly isolated and cultured cells from WT or Tg(hTNF) mice (**Figures 1G, H**). Together, these findings demonstrate a marked reduction in TNF-mediated inflammation and pathologic bone resorption in the setting of DC-STAMP deficiency. The effect of DC-STAMP on OC formation is consistent with previous reports in Dcstamp^-/-^ mice, but the marked reduction of inflammation in the setting of TNF over-production was striking. Thus, we examined potential mechanisms to explain these findings.

### Dcstamp^-/-^;Tg(hTNF) mice demonstrate increased bone density

**Figure 2:**
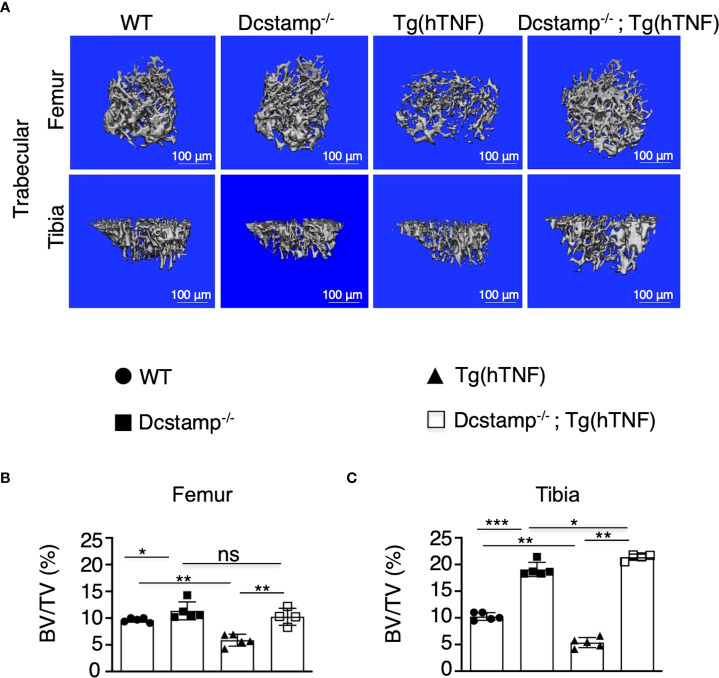
Bone density is increased in Tg(hTNF) mice lacking DC-STAMP. **(A)** Micro-CT images of femoral (upper) and tibial trabecular areas (bottom) of 5-month-old female mice show lower bone density in Tg(hTNF) mice compared with WT, Dcstamp^-/-^ and Dcstamp^-/-^; Tg(hTNF) mice. The percentage of bone volume in femur **(B)** and tibia **(C)** were considerably reduced in Tg(hTNF) mice. Data represent the mean ± SD. n = 4-5 mice per group/one back paw per mouse. ns, not statistically significant *p < 0.05, **p ≤ 0.005, ***p ≤ 0.001 (Mann-Whitney test). BV/TV= bone volume fraction.

The cell-cell fusion of osteoclast precursors to form a polykaryon is essential during the early stages of osteoclastogenesis to remove bone. DC-STAMP^-/-^ cells are primarily mononuclear and although they do undergo osteoclastogenesis, the area and depth of resorption are markedly reduced (12, 11). Of note, the effect of DC-STAMP knockout on osteoclast phenotype in a high TNF environment has not been reported. Exposure of monocytes to TNF induces a wave of calcium oscillations and subsequent induction and activation of NFATc1 that enhances osteoclastogenesis in response to RANKL (22) Thus, to determine whether the absence of DC-STAMP attenuates bone resorption during arthritis development, we evaluated bone density in the growth plate of tibias and femurs from 5-month-old Tg(hTNF) and Dcstamp^-/-^;Tg(hTNF) female mice, along with Dcstamp^-/-^ and WT controls. Consistent with the compromised generation of multinucleated OCs, micro-CT showed reduced bone volume (BV/TV; **Figures 2A–C**) in Tg(hTNF) versus Dcstamp^-/-^;Tg(hTNF) mice. These results further validate the central importance of DC-STAMP in the bone resorption phenotype characteristic of TNF-driven erosive arthritis.

### DC-STAMP modulates local and systemic inflammation induced by TNF overexpression

**Figure 3:**
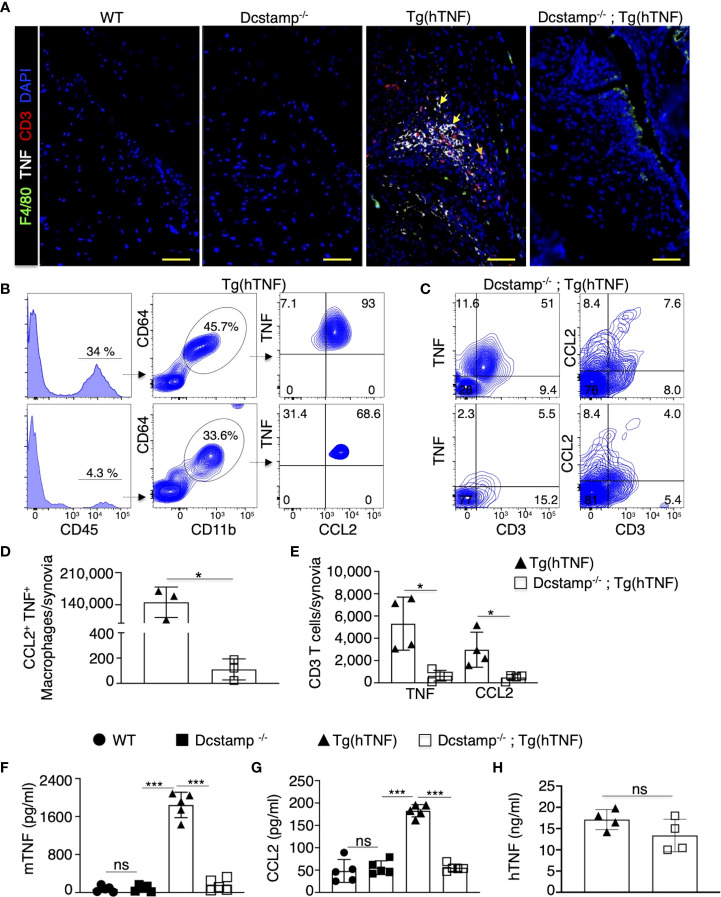
Significant decrease in local and systemic inflammation in Tg(hTNF) mice with genetic deletion of DC-STAMP. **(A)** Joint tissue sections from 5-month-old WT, Tg(hTNF), Dcstamp^-/-^ and Dcstamp^-/-^;Tg(hTNF) female mice were stained with antibodies against TNF (white), F4/80 (green) and CD3 (red). **(A)** significant decrease in the accumulation of inflammatory cells was observed in synovia from Dcstamp^-/-^;Tg(hTNF) mice. Representative 200x magnification pictures from five mice in each group were taken with a Zeiss Axioplan microscope and recorded with a Hamamatsu camera. **(B, C)** Tg(hTNF) mice demonstrated a lower frequency and total number of CCL2 and F4/80 macrophages and CD3 **(D, E)** in the synovium from the knee (n = 4; paired Student's t-test). **(F–H)** Serum from WT, Tg(hTNF), Dcstamp^-/-^ and Dcstamp^-/-^;Tg(hTNF) mice were collected at five months of age and human TNF, mouse TNF and CCL2 amounts were analyzed with the LEGENDplex TM inflammation panel assay. Murine TNF levels were significantly higher in the Tg(hTNF) compared to the mice with DC-STAMP deletion. Graphs represent mean ± SD (n = 5). *p ≤ 0.05, ***p < 0.0005, ns, not statistically significant (two-ways ANOVA and Tukey's test) **(E)** human TNF in serum was measured by ELISA, graph show similar levels of hTNF in Tg(hTNF) and Dcstamp^-/-^;Tg(hTNF) mice. Graph represents the mean ± SD (n = 4 ). ns; no significance (paired Student's t-test).

The synthesis and translocation of the transcription factor NFATc1 are crucial to coordinate bone homeostasis through its actions as the master regulator of OC formation and modulation of cytokine secretion and T cell differentiation or activation (23, 24). NFATc1 induces transcription of DC-STAMP early in osteoclastogenesis. However, studies in Dcstamp^-/-^ mice revealed that DC-STAMP regulates NFATc1 protein synthesis and nuclear translocation at later stages, supporting reciprocal regulation (12, 25). Therefore, in the absence of DC-STAMP, the recruitment of T cells, monocytes and OC precursors to the joint may be compromised in the setting of TNF-driven inflammatory arthritis. Thus, we evaluated local and systemic inflammation in Tg(hTNF) and Dcstamp^-/-^;Tg(hTNF) mice. We found a significantly greater accumulation of infiltrating CCL2^+^, TNF^+^ F4/80^+^ macrophages and CD3^+^ T cells producing CCL2 or TNF in the synovia of Tg(hTNF) compared to Dcstamp^-/-^;Tg(hTNF) mice (**Figure 3A**). We next enumerated the number of CCL2-, TNF-producing macrophages and CD3 T cells in synovial tissues from Tg(hTNF) and Dcstamp^-/-^;Tg(hTNF) mice by flow cytometry. We found that the frequency and total number of CCL2-, TNF-producing macrophages and CD3 T cells were significantly reduced in Tg(hTNF) mice lacking Dcstamp (**Figures 3B–E**). Based on the marked differences in TNF^+^CCL2^+^F4/80^+^ macrophages and CD3 T cells in the synovia of Tg(hTNF) and Dcstamp^-/-^;Tg(hTNF) mice, we next measured TNF and the monocyte-attracting chemokine CCL2 in the serum. Consistent with the decreased number of TNF^+^ and CCL2^+^ cells in the synovia, we found a significant reduction in the systemic levels of murine TNF and CCL2 in Dcstamp^-/-^;Tg(hTNF) mice (**Figures 3F, G**). However, levels of human TNF in sera from both Tg(hTNF) and Dcstamp^-/-^;Tg(hTNF) mice were similar due to the fact that human TNF is constitutively expressed in this model (**Figure 3H**). Collectively, these results suggest that DC-STAMP modulates synovial and systemic murine TNF resulting in an enhanced inflammatory response.

### DC-STAMP modulates ankle inflammation, inflammatory cell infiltration and OC function in serum-induced RA

**Figure 4:**
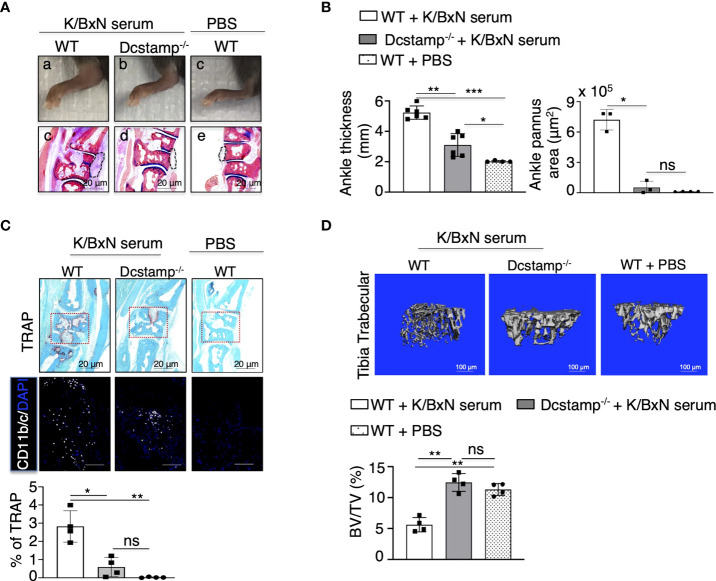
DC-STAMP modulates ankle inflammation in an acute mouse model of RA. 8-weekold C57BL/6 mice were injected, at days 0 and 3, with 150 ul of serum collected from 8-week-old K/BxN mice. Dcstamp^-/-^ mice show **(A, B)** a significant reduction in ankle thickness and pannus area, lower TRAP deposition, diminished tissue infiltration by inflammatory cells (**C** lower panel) and significantly lower bone density in the tibia **(D)** *p < 0.05, **p < 0.005, ***p < 0.0001 (Two-way ANOVA and Student's t-test). Representative 200x magnification pictures were taken with Zeiss Axioplan microscope and recorded with Hamamatsu camera. Scale bars represent 20 μm. ns, not statistically significant (paired Student's t-test).

To examine if DC-STAMP moderates the development of RA effector mechanisms, we transferred serum from K/BxN mice into WT and Dcstamp^-/-^ mice at days 0 and 3. Serum from K/BxN mice contains autoantibodies against glucose-6-phosphate isomerase that induce destructive arthritis mediated by T and B cells (26). Dcstamp^-/-^ mice showed significantly reduced inflammation along with a marked decrease in bone erosion, ankle thickness and pannus area compared to WT mice on day 12 after K/BxN serum transfer (**Figure 4A, B**). We next assessed the role of DC-STAMP in the control of local inflammation and bone resorption by visualizing CD11b^+^ monocytes and TRAP^+^ OC area in the ankles of the experimental mice. We found that in the absence of DC-STAMP, monocyte infiltration and local generation of functional multinucleated OCs were significantly decreased in Dcstamp^-/-^ compared with the monocyte infiltration and OC activity in WT mice (**Figure 4C**). In addition, trabecular bone in the tibia of WT mice that received three administrations of K/BxN serum (days 0, 3 and 26) showed lower bone volume than Dcstamp^-/-^ mice at day 30 after K/BxN serum transfer (**Figure 4D**). These findings demonstrate that DC-STAMP deficiency inhibits synovitis and bone loss in acute arthritis through inhibition of monocyte migration and OC multinucleation.

### DC-STAMP regulates macrophage migration and cytokine production

**Figure 5:**
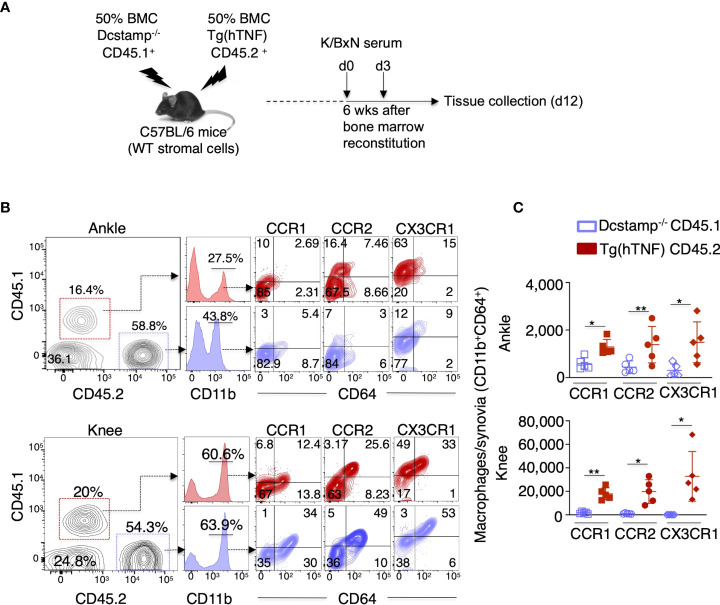
Dcstamp^-/-^ macrophages demonstrate impaired cell migration and decreased expression of chemokine receptors. **(A)** 7-week-old C57BL6 female mice were bone marrow reconstituted with 1x10^7^ CD45.1 Dcstamp^-/-^ and 1x10^7^ CD45.2 Tg(hTNF) bone marrow cells from 8-week-old mice. Six weeks after bone marrow transplant, mice were injected at days 0 and 3 with serum collected from 8-week-old K/BxN mice and sacrificed 12 days after second serum injection. Cell suspensions isolated from synovium were stained with CD45.1, CD45.2, CD11b, CD64, CCR1, CCR2 and CX3R1 antibodies. Graphs show the mean ± SD of CD45.1 or CD45.2 cell frequency **(B)** and number of CCR1, CCR2 and CX3CR1 expressing macrophages **(C)**. n = 5 mice/group. *p< 0.05, **p ≤ 0.005 (Two-way ANOVA and paired Student’s t-test).

Next, we examined if the altered immune cell joint migration observed in Dcstamp^-/-^;Tg(hTNF) mice resulted from an intrinsic defect in the immune cells or a decrease in chemo-attractant production by stromal cells. Thus, we transferred an equal mixture of BM from CD45.1 Dcstamp^-/-^ and CD45.2 Tg(hTNF) mice into lethally irradiated C57BL/6 mice (50/50 BM chimeras) to evaluate the migration of transferred cells (WT vs KO) in a WT environment in which stromal cells produce normal amounts of chemokines. We then transferred serum from the K/BxN murine arthritis model into the 50/50 BM chimeric mice at 6 weeks after reconstitution (**Figure 5A**). First, we collected blood from the chimeric mice before transfer of K/BXN serum to demonstrate the successful reconstitution of mice with CD45.1 and CD45.2 BM by flow cytometry. We confirmed the efficient reconstitution of recipient mice, which had 50% CD45.1^+^ and 50% CD45.2^+^ cells in the blood (**Supplementary Figure 1**). We then evaluated the percentage of CD45.1^+^ (KO) and CD45.2^+^ cells (WT) in the popliteal lymph nodes, spleen and synovium on day 12 after K/BxN serum transfer. The CD45.1^+^ DCSTAMP deficient cells preferentially accumulated in popliteal lymph nodes and spleen (**Supplementary Figure 1**), whereas CD45.2^+^ TNF-producing cells were significantly increased in the inflamed knee and ankle synovium of mice with acute arthritis (**Figure 5B**). Next, we quantified the number of synovia-infiltrating macrophages that express CCR1^+^ CCR2^+^ or CX3CR1^+^ receptors, which bind chemokines involved in the pathogenesis and progression of inflammatory arthritis (27). The number of macrophages expressing CCR1, CCR2 and CX3CR1 receptors in the knee and ankle synovia was significantly higher in CD45.2 Tg(hTNF) compared to CD45.1 Dcstamp^-/-^ macrophages (**Figures 5B, C**, flow cytometry gating strategy shown in **Supplementary Figure 2**). Impaired accumulation of macrophages expressing chemokine receptors and lower serum levels of CCL2 suggest that DC-STAMP is required for optimal production of chemokines that attract monocytes to the inflamed synovium.

**Figure 6:**
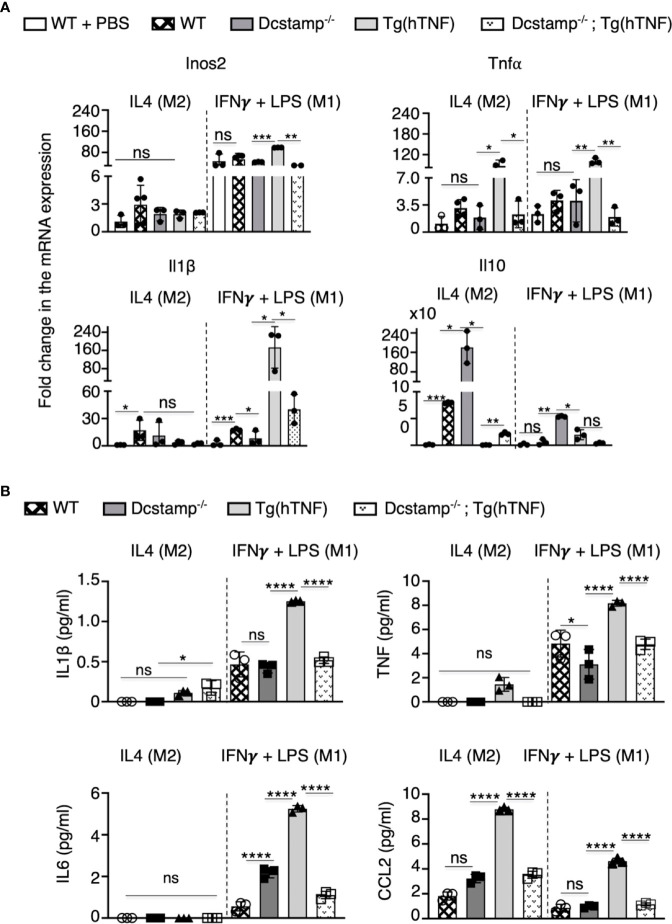
Absence of DC-STAMP decreased the expression of pro-inflammatory and antiinflammatory cytokines in macrophages from Tg(hTNF) mice. **(A)** mRNA expression for Nos2, Tnf, Il-16 and 1l-10 was estimated by RT-PCR in WT, Dcstamp^-/-^, Tg(hTNF) and Dcstamp^-/-^;Tg(hTNF) CD11b+ adherent macrophages stimulated with the M2 polarizing cytokine IL-4 or the M1 polarizing factors: IFNY + LPS. **(B)** Quantification of IL1B, TNF, IL6 and CCL2 in supernatants collected at 72 h from cultures of adherent macrophages. Graphs represent the mean ± SD (n = 3). ns, not statistically significant,*p < 0.05, **p < 0.005, ***p < 0.001, ****p < 0.0001 (Two-way ANOVA and paired Student's t-test).

To assess the effects of DC-STAMP on monocyte cytokine expression, we purified splenic CD11b^+^ adherent cells from the different experimental groups and incubated the cells in M1 or M2 polarizing conditions. Interestingly, macrophages isolated from Tg(hTNF) mice express elevated Tnf, Il1β and Inos2 levels along with decreased expression of IL10 compared to macrophages isolated from DCSTAMP^-/-^ mice. Notably, macrophages from Dcstamp^-/-^;Tg(hTNF) mice, incubated under M1 polarizing conditions, demonstrated impaired Tnf, Il1β and Inos expression, indicating DC-STAMP regulated the production of proinflammatory cytokines by M1 macrophages (**Figure 6A**). We also quantified the secretion of proinflammatory cytokines and CCL2 and found lower production of IL1β, TNF, IL6 and CCL2 by macrophages from Dcstamp^-/-^;Tg(hTNF) mice (**Figure 6B**). Thus, the absence of Dcstamp in Tg(hTNF) mice compromised both inflammatory cytokine production and macrophage migration to inflamed synovium, which indicates a dysregulated inflammatory response coupled with reduced osteoclastogenesis.

## Discussion

Given the defective cell-cell fusion of OCs and the established osteopetrosis phenotype in Dcstamp^-/-^ mice (13, 28), we investigated the hypothesis that DC-STAMP expression is essential to the pathogenesis of inflammatory arthritis. Herein, we demonstrate that DC-STAMP deficiency reduced joint swelling, bone resorption, synovitis and pannus formation in the Tg(hTNF) model of chronic inflammatory-erosive and acute arthritis in the K/BXN model. The reduced arthritic severity in Dcstamp deficient mice expressing human TNF was associated with impaired monocyte chemotaxis and cytokine production, and these monocytes exhibited an M2 phenotype. Together, these findings implicate DC-STAMP as a functional mediator of both bone homeostasis and joint inflammation with the potential to serve as a response biomarker and/or therapeutic target for inflammatory arthritis.

Osteoclast precursors (OCPs), macrophages and monocytoid dendritic cells express DC-STAMP (29), which is essential for cell-cell fusion; however this molecule does not participate in OC differentiation (30). DC-STAMP is also required for the formation of foreign body giant cells in response to GM-CSF and IL-4 (13). DC-STAMP localizes to the endoplasmic reticulum and Golgi bodies of myeloid dendritic cells and is likely involved in antigen presentation (7). As demonstrated in this study, the absence of DC-STAMP was associated with a dramatic suppression of synovitis and bone damage in well-established murine models of inflammatory arthritis. Collectively, these reports implicate DC-STAMP as a regulator of cell-cell fusion and inflammation. Thus, the marked dearth of inflammation and bone loss in our arthritis models triggered a detailed examination of cell populations in the circulation and the joint.

Signals induced by RANKL engagement, co-stimulation of the immunoreceptor tyrosine-based activation motif (ITAM), OSCAR, and triggering receptor expressed by myeloid cells (TREM)2, collectively participate in the differentiation of OC progenitors. Of particular relevance to signaling during osteoclastogenesis was our discovery of an inhibitory motif (ITIM) in the cytoplasmic tail of DC-STAMP (12). Deletion of an amino acid sequence containing this motif completely blocked cell-cell fusion and inhibited nuclear translocation of NFATc1. We proposed a model where DC-STAMP inhibits ITAM signaling *via* its ITIM which is degraded during osteoclast differentiation, permitting second signals from the ITAM motifs to sustain low level calcium signaling required for continued NFATc1 translocation (12). Thus, the differentiation and activation of OCs is the result of RANKL-RANK interaction along with secondary ITAM-ITIM signals involved in generating calcium signaling, which is analogous to mechanisms operative in inflammatory and immune cells (12, 31, 32).

In the joints of Dcstamp deficient Tg(hTNF) mice, the marked reduction in TRAP^+^ area and bone erosion paralleled previous reports in Dcstamp^-/-^ mice (28). Chronic exposure to TNF potentiates osteoclastogenesis through the induction of Ca^+2^ signaling and NFATc1 translocation (33). Thus, the reduced TRAP levels indicate that the absence of DC-STAMP overrides TNF-enhanced osteoclastogenesis. In addition, we noted a decreased frequency of CD11b^+^ cells in the synovia of the Dcstamp deficient Tg(hTNF) mice, which could be linked to decreased serum levels of CCL2 and reduced numbers of CCR1, CCR2 and CX3CR1^+^ monocytes in the inflamed synovia. Ibañez et al. found that inflammatory OCs present antigens to CD4^+^ T cells, which induce the proinflammatory cytokines TNFand IFN (34). It is well known that TNF stimulates the production of CCL2. In addition,IFNγ promotes the release of interferon induced chemokines, ligands for CXCR3 expressed on T helper one (Th1) cells (35). Thus, it is possible that by impairing the generation of multi-nucleated inflammatory OCs, DC-STAMP indirectly decreases the TNF-dependent production of CCL2 and the production of IFN-induced chemokines, affecting the recruitment of CCR2+ OC precursors and CXCR3-expressing Th1 cells. Moreover, Dcstamp knockout macrophages displayed an M2 phenotype *in vitro*, similar to the phenotype of monocytoid DC from Dcstamp^-/-^ mice (17). These findings demonstrate that DC-STAMP regulates macrophage activation and migration to inflamed synovia in acute and chronic arthritis.

Initial reports established that DC-STAMP drives cell-cell fusion and its deficiency results in mild osteopetrosis (13, 28). In addition, aged Dcstamp^-/-^ mice develop systemic autoimmunity with lymphoproliferation, splenomegaly, widespread organ inflammation and production of dsDNA autoantibodies (16). *In vitro* studies with monocytoid DC from the aged Dcstamp^-/-^ mice showed enhanced phagocytosis and immune cell activation, supporting the concept that DC-STAMP regulates phagocytosis and is essential in self-tolerance. In contrast, knocking down DC-STAMP in bone marrow dendritic cells from younger mice impaired the release of IL-6, IL-12 and TNF; and decreased IL-1β, promoting Th2 differentiation (17). Collectively, these studies indicate that DC-STAMP orchestrates monocyte signaling pathways involved in inflammation and bone remodeling, which reveals multiple potential roles for DC-STAMP to mediate the onset and/or progression of inflammatory arthritis.

With these various phenotypes associated with DC-STAMP deficiency in mind, the suppression of inflammation in the Dcstamp^-/-^;Tg(hTNF) model stands in marked contrast to the emergence of autoimmunity in aged Dcstamp^-/-^ mice. For instance, we observed that the markedly reduced synovitis correlated with low levels of CCL2 and impaired accumulation of CCR2^+^ monocytes. However, a significant barrier to interpreting DC-STAMP signaling is that its ligand is unknown, impeding the study of DC-STAMP-dependent mechanisms involved in immune cell migration and chemokine production.

We previously examined activation pathways using a photoactivation construct with deletion of the C- terminal DC-STAMP tail containing an ITIM that resulted in disrupted Ca^+2^ signaling and impaired nuclear translocation of NFATc1 (12). Thus, a potential model to explain the suppressed inflammation in Dcstamp deficient Tg(hTNF) mice is a disruption of low-level Ca^+2^ signaling resulting in altered cell differentiation and function. High levels of TNF that enhance ATP-induced Ca^+2^ transients, were unable to overcome the negative impact of DC-deficiency, presumably because DC-STAMP acts downstream on Ca^+2^ signaling through OSCAR and/or TREM pathways (11).

The findings in this study also have translational implications from several different perspectives. Regarding bone disorders, single nucleotide polymorphisms (SNPs) in the Dcstamp gene are observed in patients with Paget’s Disease (36). The OCs in this disorder are increased in number and contain up to 100 nuclei with characteristic nuclear inclusions (37). In another report, decreased methylation of DC-STAMP is observed with aging and correlates with the level of multinucleation and bone resorptive activity *in vitro* (37). Indeed, the level of OC multinucleation was associated with reduced sensitivity to zoledronic acid, raising the possibility that DC-STAMP may mark resistant pools of osteoclasts in patients with osteroporosis (38). In psoriatic arthritis, DC-STAMP surface expression on OCPs serves as a biomarker (11, 39). Our findings point to the importance of DC-STAMP as a promotor of inflammation. Importantly, future investigation into the role of DC-STAMP in rheumatoid or psoriatic arthritis is warranted to elucidate the potential utility of DC-STAMP as a therapeutic target for these prevalent diseases.

Despite the progress in understanding the role of DC-STAMP in the pathogenesis of inflammatory arthritis, our study has several limitations. Since we utilized a global knockout model of DC-STAMP deficiency, we were unable to investigate the contribution of different cell populations on arthritis or the temporal relationship of DC-STAMP deficiency at different disease stages. Lastly, while we examined DC-STAMP function in the chronic Tg(hTNF) and acute KBxN serum transfer models of inflammatory arthritis, it would also be informative to assess the impact of DC-STAMP deficiency in collagen-induced arthritis (CIA), a recognized autoimmune model of rheumatoid arthritis.

**Figure 7:**
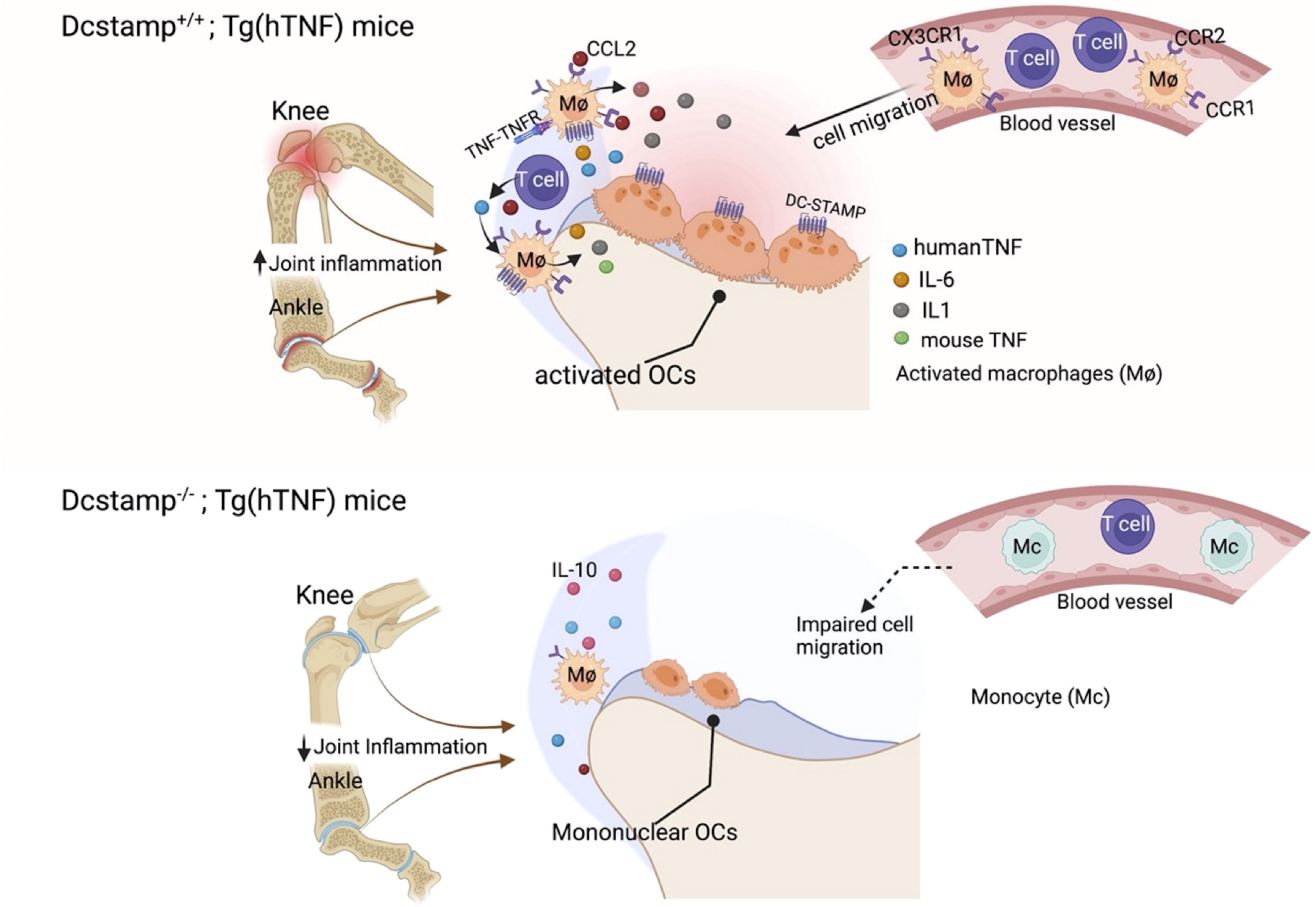
DC-STAMP enhances inflammation and bone resorption. TNF activates monocytes/DCs, which secrete pro-inflammatory cytokines and chemokines capable of recruiting innate immune cells, T cells and activating osteoclast precursors. DC-STAMP enhances TNF and CCL2 production by synovial infiltrating T cells and macrophages. In addition, bone resorption is impaired in the absence of DC-STAMP due to the inability of the OCPs to fuse and form a polykaryon. Thus, the reduction in inflammation and bone damage in Dcstamp^-/-^;Tg(hTNF) deficient is the result of amelioration of DC-STAMP driven inflammation and osteoclastogenesis.

In summary, the absence of DC-STAMP, a transmembrane molecule required for cell-cell fusion in osteoclasts and foreign body giant cells, was associated with markedly reduced synovitis and pathologic bone damage in both acute and chronic inflammatory arthritis murine models (**Figure 7**). Mechanistic studies showed diminished release of TNF by synovial infiltrating cells from DC-STAMP deficient mice and impaired cell migration. These findings will stimulate additional investigations centered on understanding the considerable promise of DC-STAMP as a novel biomarker and therapeutic target in inflammatory arthritis and metabolic bone disorders.

## Data availability statement

The original contributions presented in the study are included in the article/**Supplementary Material**. Further inquiries can be directed to the corresponding author.

## Ethics statement

The animal study was reviewed and approved by University Committee on Animal Resources (UCAR No. 102265) at the University of Rochester. UCAR@URMC.rochester.edu.

## Author contributions

Study Design: MG-H, CR. Study Conduct: MG-H, JR-M, MG-C, HK, Data analysis: MG-H, MG-C, MT. Data Interpretation: MG-H, JR-M, AA, AP. Drafting Manuscript: MG-H, CR, JR-M. Revising manuscript content: MG-H, JR-M, MG-C, HK, CR, AA. MG-H and CR take responsibility for the integrity of the data analysis. All authors contributed to the article and approved the submitted version.

## Funding

This study was supported by the National Institutes of Health: CTR (AR0169000 and AR059646), EMS (NIH R01 AR056702 and P30 AR069655), RTD (R01 AR078000) and JR-M (R01AI111914). The National Psoriasis Foundation: CTR (NPF2017DG02 ) and MLG-H (NPF851079).

## Acknowledgments

We thank Iannis E. Adamopoulos, Benjamin Korman and John Looney for the critical revision of the manuscript. Graphical figure was created with Biorender.com.

## Conflict of interest

The authors declare that the research was conducted in the absence of any commercial or financial relationships that could be construed as a potential conflict of interest.

## Publisher’s note

All claims expressed in this article are solely those of the authors and do not necessarily represent those of their affiliated organizations, or those of the publisher, the editors and the reviewers. Any product that may be evaluated in this article, or claim that may be made by its manufacturer, is not guaranteed or endorsed by the publisher.

## Abbreviations

OCPs, Osteoclast precursors; OCs, Osteoclast; RANKL, the Receptor Activator of NF-κB ligand; RANK, the receptor activator of NF-kappa B; DC-STAMP, Dendritic Cell-Transmembrane Protein; Tg(hTNF), Tumor necrosis factor transgenic; NFATc1, Transcription factor nuclear Factor of Activated T cells c1; FLK, Fibroblast Like cells; TRAP, Tartrate-Resistant Acid Phosphatase; BMDCs, Bone marrow dendritic cells; ITAM, Immunoreceptor tyrosine-based activation motif; OSCAR, osteoclast-associated receptor; TREM2, Triggering receptor expressed by myeloid cells; ITIM, inhibitory motif; M-CSF, macrophage colony-stimulating factor; Ca^2+^, calcium; SD, standard Deviation.
