# Supplementary material for: Dendritic cell-specific transmembrane protein is required for synovitis and bone resorption in inflammatory arthritis
Source: Front Immunol. 2022 Nov 7;13:1026574. doi: 10.3389/fimmu.2022.1026574 (PMC9677122; doi:10.3389/fimmu.2022.1026574)
Supplement: Supplementary file 2 [file Image_2.pdf]

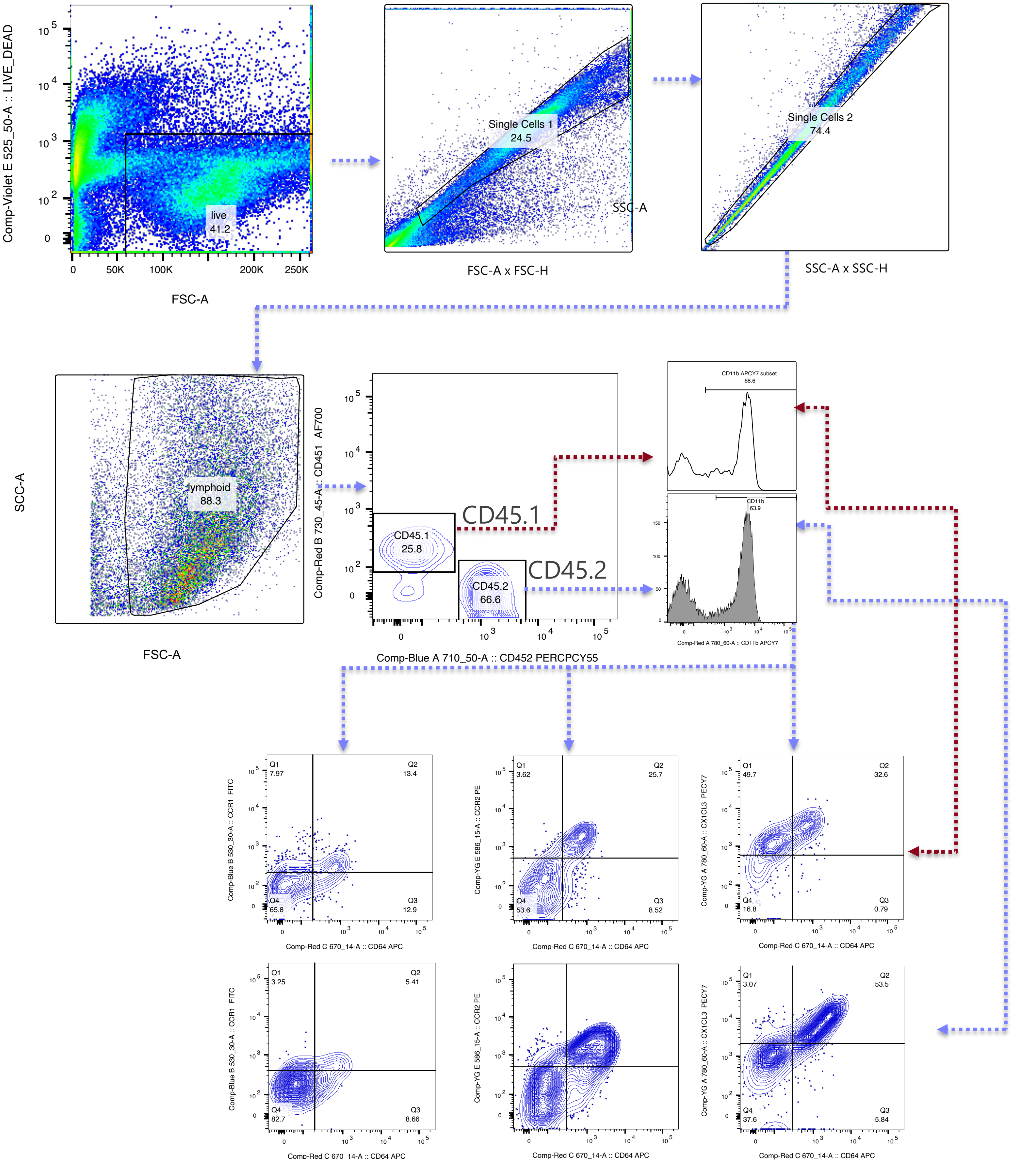


**Supplementary figure 2:** Gating strategy to analyze CCR1-, CCR2- and CX3CR-expressing macrophages isolated from knee and ankle synovium. Dead and doublets cells were excluded from the flow cytometry analysis. Leukocytes were gated based on the forward and side scatter (size and granularity), followed by a gate on CD45.1$^{+}$ or CD45.2$^{+}$ cells. We draw a gated on CD11b$^{+}$ leukocytes and select the cells based on CD64 and CCR1 or CCR2 or CX3CR1 expression.
